# Review of the Psychodinae from Mallorca, Spain, with description of *Pericoma unipennata*, sp. n. (Diptera, Psychodidae)

**DOI:** 10.3897/zookeys.577.7679

**Published:** 2016-04-05

**Authors:** Gunnar Mikalsen Kvifte, Morten Stokkan, Rüdiger Wagner

**Affiliations:** 1 Dept. of Zoology-Limnology, University of Kassel, Heinrich-Plett-Strasse 40, 34132 Kassel-Oberzwehren, Germany; 2 Dept. of Natural History, University Museum of Bergen, P.O. Box 7800, University of Bergen, 5040 Bergen, Norway; 3 Department of Biodiversity and Conservation, Instituto Mediterraneo de Estudios Avanzados (IMEDEA, CSIC-UIB), Esporles, Spain

**Keywords:** Moth flies, Balearic Isles, new species, faunistics, distribution, check list

## Abstract

We review the Psychodinae of Mallorca, recognising fifteen species based on recent collections and available literature. Previously unpublished data is presented for eleven species, of which *Neoarisemus ibericus* Wagner, 1978, *Mormia tenebricosa* (Vaillant, 1954), *Clogmia albipunctata* (Williston, 1893), *Lepiseodina rothschildi* (Eaton, 1913), *Paramormia ustulata* (Walker, 1856), *Philosepedon pyrenaicus* Vaillant, 1974 and Psychoda (Psycha) grisescens Tonnoir, 1922 are first records for Mallorca. An old record of *Pericoma trifasciata* (Meigen, 1804) is considered doubtful. *Pericoma unipennata* **sp. n** is described and illustrated based on a male collected at Deía. Distributional data are reviewed for all newly recorded species. Based on the Psychodinae fauna, the zoogeographical affinities of Mallorca are briefly discussed.

## Introduction

The Psychodidae (moth flies and sand flies) are a species-rich and widespread group of small insects mainly associated with humid habitats. The most thoroughly studied faunas of the group are found in Europe, from where more than 500 species have been described and new discoveries continue to be made (Wagner 2004, Wagner and Kvifte 2015). The most poorly studied moth fly faunas of Europe are those of the Iberian peninsula (Wagner 2001) and the Balearic islands of Spain.

The most thoroughly studied moth flies from the Balearic islands belong to the subfamily Phlebotominae, the sand flies. On Mallorca, the largest of the Balearic islands, phlebotomine sand flies have received much attention due to their significance in veterinary medicine as vectors of canine leishmaniasis. This disease is widespread and long established, to the point that some local dog breeds have evolved resistance to the parasite (Solano-Gallego et al. 2000). Four species of *Phlebotomus* and one of *Sergentomyia* have been recorded from the island, of which *Phlebotomus ariasi* Tonnoir, 1921 has been suggested as a doubtful record (Alcover et al. 2014).

Other subfamilies of Psychodidae have been studied far less. Wagner (1990) listed a single species, namely *Pericoma barbarica* Vaillant, 1955 based on Vaillant (1978). Wagner et al. (2002) further included a record of *Pericoma trifasciata* (Meigen, 1818), most likely based on a typographical error in Vaillant (1978). The *Pericoma trifasciata* record was not mentioned by Wagner (2004), who instead listed *Psychoda minuta* Banks, 1894, *Psychoda phalaenoides* (Linnaeus, 1758), Psychoda (Tineria) alternata Say, 1824 and Psychoda (Tinearia) lativentris Berdén, 1952 based on unpublished material (the latter two placed in *Tinearia* Schellenberg, 1803, treated by Wagner 2004 as a genus).

In the present study, we review existing records of Mallorcan Psychodinae and present new material for eleven species; seven of which are previously unknown from Mallorca. In addition, we describe *Pericoma unipennata* sp. n as new to science.

## Material and methods

Specimens were collected mainly by sweep netting and with aspirators from vegetation near the presumed larval habitats and preserved in 70–100% alcohol. Male specimens were sorted, dissected and mounted on slides in euparal (material in coll. ZFMK and ZMUB) or Canada balsam (material in coll. RW). Morphological terminology is according to Quate and Brown (2004) and Kvifte (2014, 2015). The „median moveable appendage“ in Kvifte et al. (2013) is here recognised as a *parameral sclerite*. Measurements are given in µm with an accuracy of 3 µm; except wing length which is given in mm to an accuracy of 100 µm.

Both literature records and new material that we have examined are included in our present checklist. Tribe-level classification is given according to Duckhouse (1987), while genus-level taxonomy is according to Wagner (2004) except where noted otherwise in the text. Species recorded as new to Mallorca are marked with an asterisk (*).

The material is deposited in the following collections:

RW
 Private collection of Rüdiger Wagner, Kassel 


ZFMK
 Alexander-König Zoologischer Forschungsmuseum, Bonn, Germany 


ZMUB
 Entomology Collections, Dept. of Natural History, University Museum of Bergen, Bergen, Norway 


## Species list

### Maruinini

#### * Neoarisemus ibericus

Taxon classificationAnimaliaDipteraPsychodidae

Wagner, 1978

##### First record from Mallorca.

Puigpunyant, 39.6167°N, 2.5167°E, 6.x.1981, H. Malicky leg. 1♂ (RW).

##### Remarks.

Previously only recorded from the type locality in northern Spain.

### Mormiini

#### * Mormia tenebricosa

Taxon classificationAnimaliaDipteraPsychodidae

(Vaillant, 1954)

##### First records from Mallorca.

Calobra, 39.85°N, 2.8°E, 9.v.1978, 90 m a.s.l., H. Malicky leg. 1♂ (RW);

Deiá, town fountain, 39.748072°N, 2.643385°E, 8.ii.2015, G.Kvifte, M. Stokkan & C. Garcia leg. 1♂ (ZFMK);

Puigpunyent, 39.62°N, 2.85°E, 12.v.1978, 200m a.s.l., H. Malicky leg., 1♂; same but 1.X.1979, 6 ♂♂; same but 6.x.1979, 6♂♂ (all RW);

South slope of Piug Major, north of Soller, 39.783°N, 2.767°E, 7.–9.v.1978, 700 m a.s.l., H. Malicky leg. 2♂♂ (RW).

##### Remarks.

Previously known from France, mainland Spain, Italy, Morocco and Algeria (Vaillant 1974). The generic and subgeneric classification of Mormiina is unstable and requires revision; therefore the placement in *Mormia* may change.

### Paramormiini

#### * Clogmia albipunctata

Taxon classificationAnimaliaDipteraPsychodidae

(Williston, 1893)

##### First records from Mallorca.

Esporles, IMEDEA research center, 39.666438°N, 2.580863°E, 13.iv.2015, M. Stokkan leg. 1♀ (ZMFK);

E of Puigpunyent, 39.616667°N, 2.85°E, 1.x.1979, 200m a.s.l., H. Malicky leg. 1♀; same but 6.x.1979, 4♀♀ (all RW).

##### Remarks.

A widespread near-cosmopolitan species, first recorded from Spain by Tonnoir (1920) under the synonym *Telmatoscopus meridionalis* (Eaton, 1894). Its biology is summarised in Boumans et al. (2009) and Ježek et al. (2012); for a review of its taxonomy see Ibañez-Bernal (2008).

#### * Lepiseodina rothschildi

Taxon classificationAnimaliaDipteraPsychodidae

(Eaton, 1912)

##### First record from Mallorca.

SW Pollensa, 39.8833°N, 2.9833°E, 3–5.x.1981, H. Malicky leg. 1♂ (RW).

##### Remarks.

Placed in *Lepiseodina* Enderlein, 1937 (type species *Psychoda tristis* Meigen, 1830) by Ježek (1990) because of its asymmetric genitalia. The placement in *Clogmia* by Ježek (1984), Vaillant (1989) and Wagner (2004) may be a valid option to consider for the future when further character systems are explored.

Previous records are from Austria, Belgium, Czech Republic, France, Germany, Great Britain, Ireland and Slovakia (Oboňa and Ježek 2012).

#### * Paramormia ustulata

Taxon classificationAnimaliaDipteraPsychodidae

(Walker, 1856)

##### First records from Mallorca.

Banyalbufar, 39.691635°N, 2.514367°E, 25.x.2012, G.Kvifte leg. 1♂1♀ (ZMUB);

Pond west of Cala Figuera, 39.335635°N, 3.152597°E, 11.ii.2015, G.Kvifte leg, 1♂ (ZFMK).

##### Remarks.

A widespread species or complex of species occurring in the Holarctic region. Ježek and Yağci (2005) list occurences from the following countries: Afghanistan, Algeria, the Azores, Belgium, Bosnia-Herzegovina, Bulgaria, the Canary Islands, China, Corsica, Czech Republic, Denmark, France, Germany, Great Britain, Greece, Hungary, Iran, Ireland, Israel, Italy, Macedonia, Madeira, Mongolia, Morocco, the Netherlands, Olanda island, Poland, Romania, Sardinia, Serbia, Slovakia, Slovenia, Spain, Sweden, Switzerland, Tunisia, Turkey and the USA.

### Pericomaini

#### Pericoma unipennata

Taxon classificationAnimaliaDipteraPsychodidae

Kvifte, Stokkan & Wagner sp. n.

http://zoobank.org/14619D1C-5A7E-4197-B99C-2C004DE54E83

##### Type material.

Holotype male. Deiá, town fountain, 39.748072°N, 2.643385°E, 8.ii.2015, G.Kvifte, M. Stokkan & C. Garcia leg (ZMUB).

**Diagnosis.** *Pericoma unipennata* can be separated from all other *Pericoma* species on the presence of one feather-tipped and four spatulate tenacula on each surstylus, as well as the following combination of characters: parameral sheath with shallow U-shaped apical indentation 1/6th as deep as width of parameral appendage base, gonostyle with narrow distal part 1/4th as long as broad basal part, distiphallic spatula with sclerotized side margins not converging apically, parameral appendage concave at lateral sides with distal 1/8th protruding over distiphallic spatula.

##### Description.

**Figure 1.**
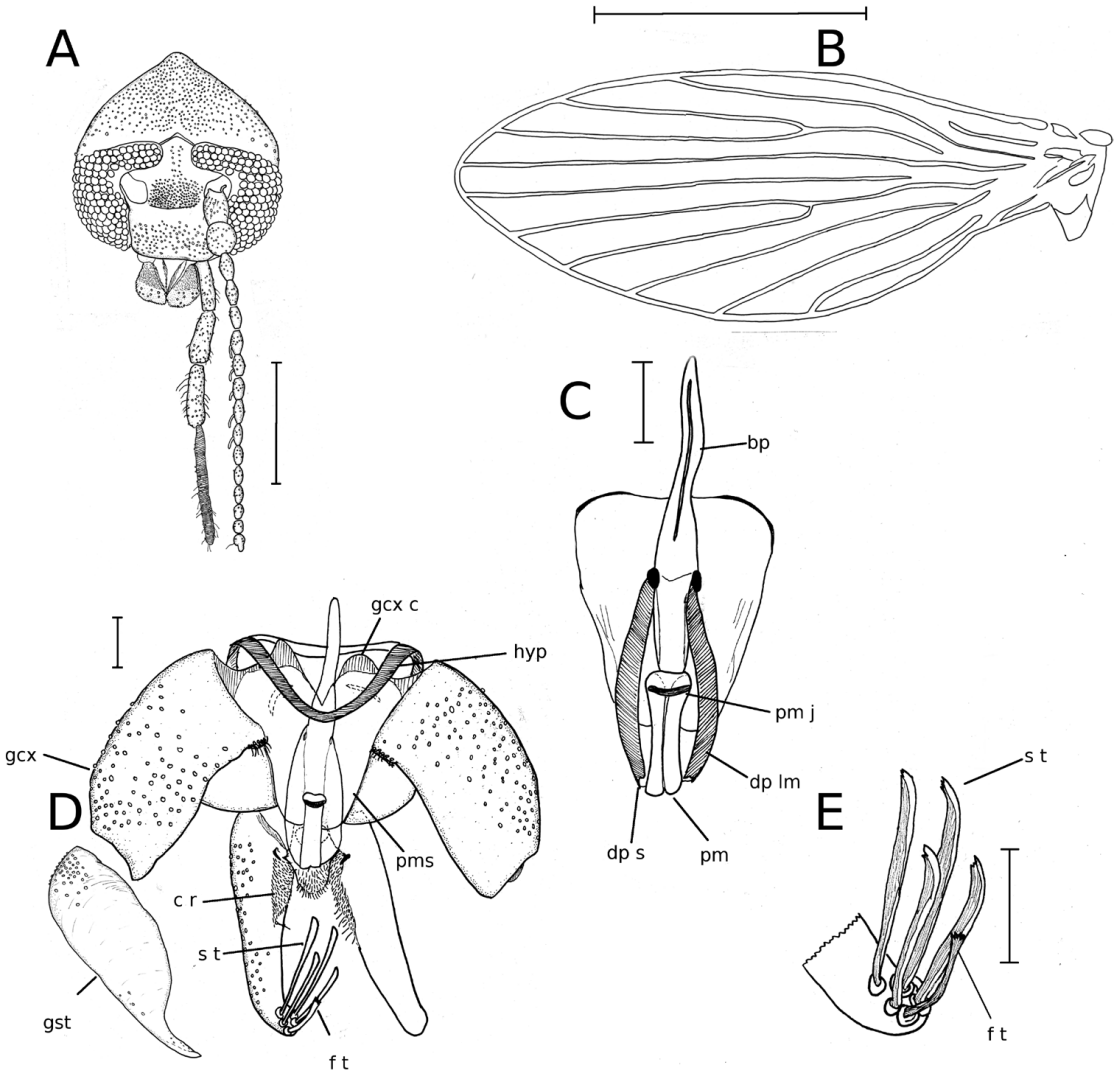
*Pericoma unipennata* sp. n., holotype male. **A** Head **B** Wing **C** Aedeagus **D** Male genitalia **E** Tenacula. The following abbreviations are used: bp – basiphallus, c r – cercal region, dp lm – distiphallic lateral margin, dp s – distiphallic spatula, f t – feathery tenacula, gcx – gonocoxite, gst – gonostyle, hyp – hypandrium, pm – parameral sclerite, pm j – parameral joint, pms – parameral sheath, s t – spatuliform tenacula.

Male (n=1). Head (Fig. 1A) longer than wide; vertex rounded with posterior lobe pointed, about half length of head; eyebridge comprising four rows of facets, separated by five facet diameters; interocular suture broadly V-shaped with median swelling; 7 supraocular setae present, 3 on ventral side and 4 on dorsal side; frontal scar patch inversely T-shaped, reaching level of uppermost facet row of eyebridge; clypeus subrectangular, weakly concave at anterior margin, densely setose, not protruding in front of level of eyes; palp with 4th segment fleshy, corrugated, length of palpus segments 93 : 106 : 129 : 231; labellum fleshy, micropilose with 11 larger setae; antennae with 14 fusiform flagellomeres; scape cylindrical, pedicel elongate globular to barrell-shaped; ascoids present on flagellomeres 4-8; flagellomere 14 with apiculus about as long as base of segment; length of antennal segments 69 : 63 : 48 : 45 : 45 : 42 : 42 : 42 : 42 : 42 : 36 : 36 : 33 : 27 : 24 : 36.

Thorax without accessory organs; mesonotum and scutellum covered in setae alveoli except on lateral margins; anepisternum and laterotergite covered with setae alveoli; coxae and trochanters with dorsal and ventral stripes of setae alveoli, mid coxa with apicoanterior setose projection; legs without special features;

Wing (Fig. 1B) 2.3 mm long, subovate, membrane without setation or infuscation; radial fork very slightly distad of medial fork, both slightly distad of CuA_2_; C with two breaks; Sc weakly curved towards C; apical section of R_2_ curved anteriorly; wing apex between R_4_ and R_5_; medial fork incomplete; apical section of CuA_2_ curved posteriorly; jugum obtuse;

Genitalia (Fig. 1C, D) with hypandrium of even width; aedeagus (Fig. 1D) with basiphallus compressed laterally; distiphallus consisting of two phallomeres forming a spatula with sclerotized lateral margins, lateral margins not converging distally; parameral sheath fused with gonocoxites basally, distally narrowing to nearly half its width at base; distally with narrow median incision; parameral sclerite meeting distal section of basiphallus, jointed to parameral sheath with slightly curved transverse band-like sclerite; parameral sclerite half length of distiphallus, consisting of two fused sclerites which are concave laterally, median suture complete; gonocoxites (Fig. 1C) stout, reniform, parabasal process absent but setose field present near connection with parameral sheath; gonocoxal condyles apparently fused with parameral sheath, gonostyle (Fig. 1C) with basal four 5ths liver-shaped, stout, distal 5th pointed, weakly sinous; epandrium slightly wider than long, expanding distally, number of apertures not discernable in specimen, carrying sparse hairs on ventral surface; surstyli about as long as epandrium, with lateral inner third more weakly sclerotized and densely micropilose; apex of surstylus with five aseriate tenacula of which one apical is feathery at the apex, four subapical tenacula spatulate; hypoproct obtusely isosceles trapezoid, micropilose; epiproct oval, densely pilose.

##### Etymology.

*unipennata* = with one feather; refers to the presence of a single feather-tipped and four spatulate tenacula on its surstyli.

##### Biology.

The specimen was collected at a spring with many bryophytes growing in a seepage stream. Bryophyte material was collected and extracted, however, no psychodid larvae or pupae were found.

#### Pericoma barbarica

Taxon classificationAnimaliaDipteraPsychodidae

Vaillant, 1955

##### New records.

Calobra, 39.85°N, 2.8°E, 9.v.1978, 90m a.s.l., H. Malicky leg. 1 ♂ (RW);

Deiá, town fountain, 39.748072°N, 2.643385°E, 8.ii.2015, G.Kvifte, M. Stokkan & C. Garcia leg. 3♂♂ (ZMUB);

Esporles, Torrent de San Vic, 39.670459°N, 2.569193°E, 11.ii.2015, G.Kvifte leg. 1♂ (ZMFK);

Puigpunyent, 39.616667°N, 2.85°E, 15.v.1978, 200m a.s.l., H. Malicky leg. 12 ♂♂; same but 1.x.1979, 5 ♂♂; same but 6.x.1979, 2 ♂♂ (all RW);

South slope of Piug Major, north of Soller, 39.783333°N, 2.766667°E, 7.–9.v.1978, 700 m a.s.l., H. Malicky leg. 11♂♂ (RW).

##### Literature record.

Vaillant (1978).

##### Remarks.

This species was described from Algeria and subsequently recorded from Mallorca by Vaillant (1978). It also occurs in mainland Spain and in North Africa (Vaillant 1978).

#### Pericoma trifasciata

Taxon classificationAnimaliaDipteraPsychodidae

(Meigen, 1818)

##### Literature record.

Wagner et al. (2002).

##### Remarks.

Vaillant (1978) wrote “[*Pericoma*] *trifasciata* ist ebenfalls gemein auf der Mallorca-Insel [...]” (“*trifasciata* is also common on the island of Mallorca”) in the paragraph summarizing the geographical distribution of *Pericoma barbarica* Vaillant, 1955; this is probably a lapsus as Mallorca is not mentioned in the species account for *Pericoma trifasciata*. No other records are available in the literature prior to the listing by Wagner et al. (2002), which was based on Vaillant (1978). In Wagner (2004) and the present paper we deem the records from the Baleares to be doubtful and in need of verification through examination of specimens.

### Psychodini

#### * Philosepedon pyrenaicus

Taxon classificationAnimaliaDipteraPsychodidae

Vaillant, 1974

##### First records from Mallorca.

Deiá, town fountain, 39.748072°N, 2.643385°E, 8.ii.2015, G.Kvifte leg. 1♂ (ZMFK);

Esporles, town fountain, 39.669519°N, 2.576953°E, 10.ii.2015, G.Kvifte leg. 1♂ (ZMFK);

Palma de Mallorca, 39.570725°N, 2.641432°E, 7.ii.2015, G.Kvifte leg. 3♂1♀ (2♂ ZMFK, 1♂1♀ ZMUB).

##### Remarks.

The males recorded here were identified as *Philosepedon pyrenaicus* according to the key in Vaillant (1974) because of the eyes separated by three facet diameters, the wing “mittwinkel” 105° and the left phallomere of the aedeagus being longer than the right. However, the specimens differ in that the 1st flagellomere is longer than the combined length of the scape and pedicel, and the eyebridge is two facet rows wide at several points. We deem these differences not to be taxonomically meaningful, as the differences in proportions and eyebridge composition could be interpreted as interspecific variation.

#### Psychoda (Psychodocha) cinerea

Taxon classificationAnimaliaDipteraPsychodidae

Banks, 1894

##### Literature record.

Wagner (2004)

##### New records.

Cala Figuera, 39.332245°N, 3.166440°E, 11.ii.2015, G. Kvifte leg. 2♂♂ (ZMFK);

Deiá, town fountain, 39.748072°N, 2.643385°E, 8.ii.2015, G.Kvifte, M. Stokkan & C. Garcia leg. 1♂ (ZMUB);

Esporles, Torrent de San Vic, 39.670459°N, 2.569193°E, 10.ii.2015, G.Kvifte leg. 1♂ (ZMFK).

#### Psychoda (Psychodocha) gemina

Taxon classificationAnimaliaDipteraPsychodidae

(Eaton, 1904)

##### Literature record.

Wagner (2004)

#### Psychoda (Psychoda) phalaenoides

Taxon classificationAnimaliaDipteraPsychodidae

(Linnaeus, 1758)

##### Literature record.

Wagner (2004).

#### * Psychoda (Psycha) grisescens

Taxon classificationAnimaliaDipteraPsychodidae

Tonnoir, 1922

##### First records from Mallorca.

Esporles, Torrent de San Vic, 39.670459°N, 2.569193°E, 11.ii.2015, G.Kvifte leg. 1♂.

##### Remarks.

Recorded from Algeria, Austria, Belgium, Bosnia-Herzegovina, Czech Republic, Denmark, the Faroe Islands, France, Germany, Great Britain, Greece, Hungary, Ireland, Italy, Morocco, the Netherlands, Norway, Slovakia, Slovenia, Sweden, Tunisia and Turkey (Andersen 1999, Ježek 2004, Ježek and Yağci 2005).

#### Psychoda (Psychodula) minuta

Taxon classificationAnimaliaDipteraPsychodidae

Banks, 1894

##### Literature record.

Wagner (2004).

##### New record.

Esporles, Torrent de San Vic, 39.670459°N, 2.569193°E, 10.ii.2015, G.Kvifte leg. 1♂.

#### Psychoda (Tinearia) alternata

Taxon classificationAnimaliaDipteraPsychodidae

+Say, 1824

##### Literature record.

Wagner (2004).

##### New record.

Esporles, town fountain, 39.669519°N, 2.576953°E, 10.ii.2015, G.Kvifte leg. 1♀ (ZMFK).

##### Remarks.

*Tinearia* Schellenberg, 1803 was treated as a genus by Wagner (2004) following Ježek (1977). This species group is undoubtedly monophyletic, however based on morphology and some analyses of molecular data it appears to be deeply nested within *Psychoda* s.l. (Vaillant 1990, Espindola et al. 2012, Kvifte and Andersen 2012, Kvifte unpubl.). Inclusion of *Tinearia* as a subgenus within *Psychoda* would thus allow ease of identification of monophyletic units, whereas recognising *Tinearia* as a separate taxon would render *Psychoda* paraphyletic. For a discussion of the monophyly criterion in supraspecific taxonomy, see Komarek and Beutel (2006).

#### Psychoda (Tinearia) lativentris

Taxon classificationAnimaliaDipteraPsychodidae

Berdén, 1952

##### Literature record.

Wagner (2004)

##### Remarks.

For genus taxonomy, see remarks under *Psychoda alternata* above.

## Discussion

The material examined in the present study was collected opportunistically and does not reflect the diversity of suitable habitats on Mallorca. Nevertheless, a few preliminary conclusions about the diversity and zoogeographic affinities of the fauna can be made.

Most of the species encountered are widespread European, Holarctic or even cosmopolitan species (*Clogmia albipunctata*, *Paramormia ustulata*, *Psychoda* spp. and arguably *Lepiseodina rothschildi*). Four species appear to have more limited distributions as local West Mediterranean elements, namely *Neoarisemus ibericus*, *Mormia tenebricosa*, *Pericoma barbarica* and *Philosepedon pyrenaicus*. A single species, *Pericoma unipennata* sp. n., has yet to be recorded outside of Mallorca but may have been overlooked elsewhere.

Our records of *Neoarisemus ibericus* and *Philosepedon pyrenaicus* are the first since the original descriptions, which in both cases were based on very few specimens collected in Northern Spain: Montes Universales and the Pyrenees respectively. The records from Mallorca represent a major range extension for both species, suggesting them to be more widespread than previously expected and that they may have been overlooked elsewhere.

Both *Mormia tenebricosa* and *Pericoma barbarica* have similar distribution patterns; occurring on the north and south coasts of the West Mediterranean. Both species appear widespread in North Africa, having been recorded from Morocco, Algeria and Tunisia (Vaillant 1974, 1978, Wagner 1987). They differ in their European distributions, with the range of *Pericoma barbarica* extending northeast to the Pyrenees and *Mormia tenebricosa* reaching the Western Alps in southern France and Italy.

Vaillant (1974) mentions consistent minor morphological differences between adult males of *Mormia tenebricosa* in the North African and European populations, but refrained from using them to delimit species. We consider that these differences warrant further study as they may be evidence of cryptic species or ongoing speciation; DNA sequences will be useful in illuminating this question.

*Pericoma unipennata* sp. n. appears to be a member of the Mediterranean *Pericoma modesta* Tonnoir, 1922 species group as defined by Vaillant (1978, p. 226). In this group, *Pericoma modesta* has a wide Mediterranean distribution whereas *Pericoma alhambrana* Vaillant, 1978, *Pericoma graecica* Vaillant, 1978 and *Pericoma motasi* Vaillant, 1978 are localized endemics in Southern Spain, Southern Balkan and the Romanian Carpathian mountains, respectively. The Psychodidae of the Iberian peninsula are too incompletely known to tell whether *Pericoma unipennata* sp. n. is an island endemic of the Balearic islands or whether it is more widespread in the Mediterranean region.

## Supplementary Material

XML Treatment for Neoarisemus ibericus

XML Treatment for Mormia tenebricosa

XML Treatment for Clogmia albipunctata

XML Treatment for Lepiseodina rothschildi

XML Treatment for Paramormia ustulata

XML Treatment for Pericoma unipennata

XML Treatment for Pericoma barbarica

XML Treatment for Pericoma trifasciata

XML Treatment for Philosepedon pyrenaicus

XML Treatment for Psychoda (Psychodocha) cinerea

XML Treatment for Psychoda (Psychodocha) gemina

XML Treatment for Psychoda (Psychoda) phalaenoides

XML Treatment for Psychoda (Psycha) grisescens

XML Treatment for Psychoda (Psychodula) minuta

XML Treatment for Psychoda (Tinearia) alternata

XML Treatment for Psychoda (Tinearia) lativentris
